# Synthesis of New Chitosan from an Endemic Chilean Crayfish Exoskeleton (*Parastacus Pugnax*): Physicochemical and Biological Properties

**DOI:** 10.3390/polym13142304

**Published:** 2021-07-14

**Authors:** César Burgos-Díaz, Mauricio Opazo-Navarrete, José Luis Palacios, Tamara Barahona, Yohanna Mosi-Roa, Fresia Anguita-Barrales, Mariela Bustamante

**Affiliations:** 1 Agriaquaculture Nutritional Genomic Center (CGNA), Temuco 4780000, Chile; mauricio.opazo@cgna.cl (M.O.-N.); tamara.barahona@gmail.com (T.B.); yohanna.mosi@cgna.cl (Y.M.-R.); fresia.anguita@cgna.cl (F.A.-B.); 2 Centro de Estudios en Ciencia y Tecnología de los Alimentos (CECTA), Universidad de Santiago de Chile, Santiago 9170201, Chile; jose.palacios@usach.cl; 3 Department of Chemical Engineering, Scientific and Technological Bioresource Nucleus (BIOREN), Universidad de La Frontera, Temuco 4780000, Chile; mariela.bustamante@ufrontera.cl

**Keywords:** chitin, chitosan, biopolymer, *Parastacus pugnax*, antibacterial properties, antioxidant properties

## Abstract

Chitin is one of the most abundant natural polysaccharides in the world and it is mainly used to produce chitosan by a deacetylation process. In the present study, the extraction of chitin and chitosan from the *Parastacus pugnax* (*P. pugnax*) crayfish exoskeleton was studied for the first time. Thus, the *P. pugnax* crayfish exoskeleton was converted to chitosan following the steps of depigmentation, deproteinization, and deacetylation. The produced chitosan (Chitosan-CGNA) was characterized in terms of the protein content, solubility, degree of deacetylation, viscosity, molecular weight, FTIR, SEM, XRD, antimicrobial, and antioxidant activity. The results showed that the obtained chitosan had a high degree of deacetylation (91.55%) and a medium molecular weight (589.43 kDa). The antibacterial activity of the chitosan was tested against bacterial strains relevant for the food industry and the lowest minimum inhibitory concentration (MIC) values were evidenced with *Salmonella tiphymurium* (*S. typhimurium*), *Staphylococcus aureus* (*S. aureus*), *Enterococcus faecalis* (*E. faecalis*) and *Listeria. Monocytogenes* (*L. monocytogenes*). Moreover, the Chitosan-CGNA showed an effect on DPPH radical scavenging activity, and its antioxidant activity was dependent on concentration and deacetylation degree. These results suggest that *P. pugnax* exoskeleton could be an excellent natural source for the production of chitosan with potential applications in the health system, and to prevent infections associated with pathogens strains.

## 1. Introduction

The burrowing crayfish (*Parastacus pugnax*) is endemic to central-southern Chile and is found continuously between the Aconcagua River in the Valparaíso Region and the town of Nehuentué in La Araucanía Region, Chile [1]. *Parastacus pugnax* is one of six species of the family Parastacidae inhabiting the fresh rivers of Chile, which supports the greatest extraction effort for human consumption [2]. Moreover, *Parastacus Pugnax* is a very valuable natural resource for smallholder farmers from southern Chile (e.g., La Araucanía), which is exploited in a sustainable way for consumption and commercialization by local communities.

During the *Parastacus Pugnax* crayfish processing and consumption, a large number of by-products such as the exoskeleton, skin, and viscera are produced and usually discarded. The improper disposal of these by-products can result in environmental problems such as unpleasant odor and sedimentation of minerals in landfill [3]. Hence, conversion of the *P. pugnax* shrimp exoskeleton waste material into valuable added products is a great opportunity to mitigate environmental problems. In addition, the valorization of fishery discarded by-products has received much attention due to the increasing awareness of its economic potential and environmental impacts [4].

In recent years, there has been a growing interest in developing natural alternatives to synthetic polymers obtained from renewable resources to develop environmentally friendly materials. Among these biopolymers, chitosan has attracted the attention of both scientists and industry due to its numerous potential applications in different industrial fields [5]. Chitosan is the only naturally occurring cationic polysaccharide. It is derived from the alkaline deacetylation of chitin, which is the second most abundant biopolymer after cellulose [6]. Chitin is naturally present as a structural element in the exoskeletons of a wide range of eukaryotic organisms like crustaceans, insects, and cell walls of fungi, and the second most abundant biopolymer next to cellulose on the planet [7].

Recently, there has been great interest in the extraction of chitosan from crustacean by-products for nutraceutical and food packing applications [8]. This can be explained by its percentage of chitin (between 10 and 25%, dried weight basis) in the crustacean shell or exoskeleton, which can be capitalized upon in the production of this economically viable natural macromolecule [9]. However, to date, there have been no studies about the extraction, characterization and properties of chitosan from the endemic Chilean crayfish (*P. pugnax*) exoskeleton.

Chitosan is considered a bioactive polymer with a wide variety of applications due to its biocompatibility, antibacterial activity, non-toxicity, ease of modification, and biodegradability [10]. Therefore, chitosan has been used in a wide variety of industrial fields such as biomedicine, cosmetology, papermaking, food industry, wastewater treatment, agriculture, textile industry and pharmaceutical applications, among others [5,10].

Regarding biological properties, chitosan has been found to possess antioxidant, hypocholesterolemic and antibacterial activities. The biological activities of chitosan are related to its molecular size, deacetylation and sources [11]. For instance, the antioxidant activity of chitosan and its derivatives has attracted great attention. Antioxidant activity is one of the well-known functionalities of chitosan and many studies have shown that chitosan inhibits the reactive oxygen species (ROS) and prevents lipid oxidation in food and biological systems [11].

Additionally, several studies have suggested that the antimicrobial activity of chitosan is a consequence of its cationic nature. The electrostatic interaction between positively charged R–N(CH_3_)_3_^+^ sites and negatively charged microbial cell membranes is predicted to be responsible for cellular lysis and is assumed to be the main antimicrobial mechanism [12].

The deacetylation degree (DD) of chitosan is one of the most important factors influencing both chemical (solubility, flexibility, polymer conformation, viscosity, crystallinity, high surface area, porosity, tensile strength, conductivity, photoluminescence) and biological properties (biodegradability, biocompatibility, mucoadhesion, hemostatic, analgesic, adsorption enhancer, antimicrobial, anticholesterolemic and antioxidant), which vary with processing conditions [13].

Consequently, this study proposed for the first time the extraction of chitosan from the exoskeleton of an endemic Chilean river crayfish (*P. pugnax*). Thus, the chitosan sample was characterized regarding their physicochemical and biological properties. Regarding biological properties of chitosan, these were studied in terms of antibacterial activity against strain bacteria of food industrial relevance and antioxidant activity.

## 2. Materials and Methods

### 2.1. Materials

The *Parastacus pugnax* crayfish exoskeletons were provided by “Cooperativa Agricola Cacike Juan Currimil de Lolocura Ltd.a.” (Carahue, La Araucanía, Chile). Commercial chitosan was purchased from a local supplier (Citrex, La Cruz, Chile) and used as a control in this study. NaOH, HCl, ethanol, NaCl, ascorbic acid and acetic acid were acquired from Sigma-Aldrich (São Paulo, Brazil), while trichloroacetic acid, Trolox reagent, fluorescein sodium salt, Bradford reagent, Methyl orange reagent were purchased from Merck (Darmstadt, Germany). Na-phosphate buffer was purchased from J.T. Baker (Deventer, The Netherlands); AAPH from Cayman Chemical (Ann Arbor, MI, USA); DPPH reagent from Calbiochem (Darmstadt, Germany); Lactic acid from Sabores (Santiago, Chile); L-Ascorbic acid from Mallinckrodt (Phillipsburg, NJ, USA); Na-phosphate buffer from J T Baker (Deventer, The Netherlands); and technical Acetone from HES (Santiago, Chile).

### 2.2. Extraction of Chitosan from Parastacus Pugnax Crayfish Exoskeleton

**Figure 1:**
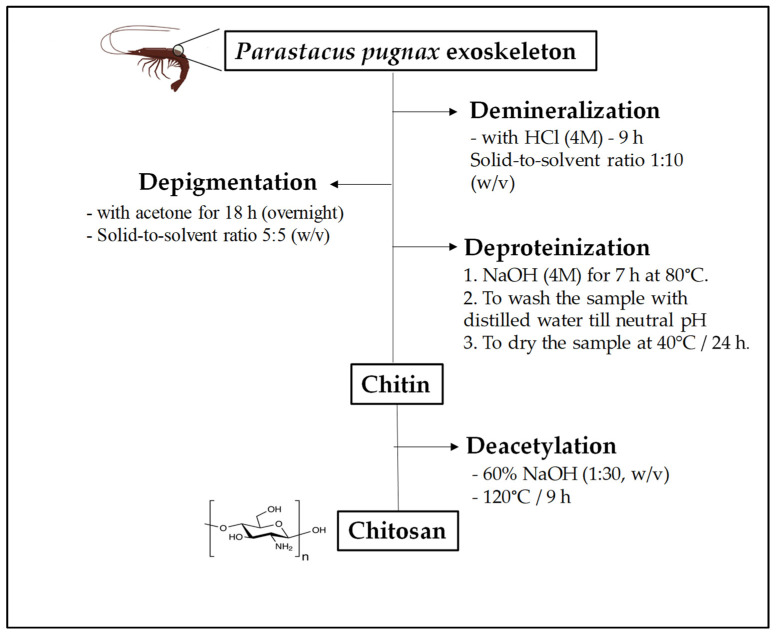
Diagram of production of chitosan from *Parastacus pugnax* exoskeleton.

The extraction of chitosan from *P. pugnax* crayfish exoskeleton was performed following the method described by Mohan et al. [3] with some modifications. Four steps were applied for chitosan preparation, including (i) demineralization, (ii) depigmentation, (iii) deproteinization and (iii) deacetylation (Figure 1). The ground *P. pugnax* exoskeleton powder was demineralized with HCl (4 M) for 9 h with a solid-to-solvent ratio of 1:10 (*w*/*v*), and then depigmented with acetone for 18 h (overnight). After that, the sample was deproteinized with NaOH (4 M) for 7 h at 80 °C. After washing with distilled water until a neutral pH was achieved, chitin powder was dried at 40 °C in an oven for 24 h. Deacetylation of the chitin was achieved by reacting the chitin with 60% NaOH (1:30, *w*/*v*) at 120 °C for 9 h. Finally, the sample was washed with distilled water until it reached a neutral pH, and the filtrate was dried at 40 °C for 24 h.

### 2.3. Determination of Intrinsic Viscosity and Molecular Weight (Mw)

The molecular weight of Chitosan-CGNA and commercial CGNA were determined by using the Ubbelohde Dilution Viscometer (Cannon-Ubbelohde, State College, PA, USA) with a capillary size of 0.58 nm. Thus, 100 mg of chitosan was dissolved in 10 mL of the mixture solution of CH_3_COOH (0.1 M) and NaCl (0.2 M). The intrinsic viscosity was measured by the intercept between the Huggins (reduced viscosity, η sp/C~C) and Kraemer (relative viscosity, η rel/C~C) plots when the concentration was 0. The intrinsic viscosity is related to molecular weight. Therefore, the molecular weight was calculated by using the Mark–Houwink Equation as follows:

(1) $$\left[\eta \right]=k{\left(\mathrm{Mw}\right)}^{\alpha }$$

where [η] is intrinsic viscosity, and K and α are constants that are characteristic for a particular polymer–solvent system (K = 1.81 × 10^−3^ and α = 0.93, respectively). “Mw” is the average molecular weight.

### 2.4. Determination of Deacetylation Degree (DD)

The DD of the chitosan was determined using an acid-base titration with NaOH (0.1 M). First, 400 mg of dry chitosan was dissolved in 30 mL of 0.1 M hydrochloric acid solution. Methyl orange reagent was used as an indicator and a small amount was applied. The DD is equivalent to the percentage of amino groups (%NH_2_) present in the chitosan polymer chain. The DD was calculated by the following equation:

(2) $$\left[{\%\mathrm{NH}}_{2}\right]=\left[\left(C_{1}\times V_{1}-C_{2}\times V_{2}\right)\times 0.016\right]/\left[G\left(100-W\right)\right]\times 100$$

where $C_{1}$ = Concentration HCl; $V_{1}$ = Volume of HCl; $C_{2}$ = Concentration NaOH; $V_{2}$ = Volume NaOH; G = sample weight; W = weight of moisture content.

### 2.5. Determination of Protein, Nitrogen and Ash Content of Chitosan

The protein content of chitosan was determined by the Bradford method [14]. For this, a Bradford reagent (Merck, Darmstadt, Germany) was used. The absorbance values were measured at 595 nm and the assay was performed in microplate wells. The protein concentration of the sample was calculated using the equation of the standard curve. Nitrogen content was determined by Dumas combustion method (AOAC 930.03). The ash content of chitosan samples was gravimetrically estimated after the pyrolysis of 1 g in a muffle at 800 °C for 5 h. This procedure was done in triplicate and the mean ash content was calculated.

### 2.6. Determination of Solubility (%) of Chitosan

Chitosan was dissolved in 1% acetic acid solution and after centrifugation (Eppendorf 5810R, Hamburg, Germany), the undissolved particles were separated and dried in an oven and weighed. The solubility of the obtained chitosan was calculated from the equation:

(3) $$\%\;\mathrm{Solubility}:\frac{\left(\mathrm{initial}\;\mathrm{weight}\;\mathrm{of}\;\mathrm{tube}+\mathrm{chitosan}\right)-\left(\mathrm{final}\;\mathrm{weight}\;\mathrm{of}\;\mathrm{tube}+\mathrm{chitosan}\right)}{\left(\mathrm{initial}\;\mathrm{weight}\;\mathrm{of}\;\mathrm{tube}+\mathrm{chitosan}\right)-\left(\mathrm{initial}\;\mathrm{weight}\;\mathrm{of}\;\mathrm{tube}\right)}\times 100$$

### 2.7. Potentiometric Titration of Chitosan

**Figure 2:**
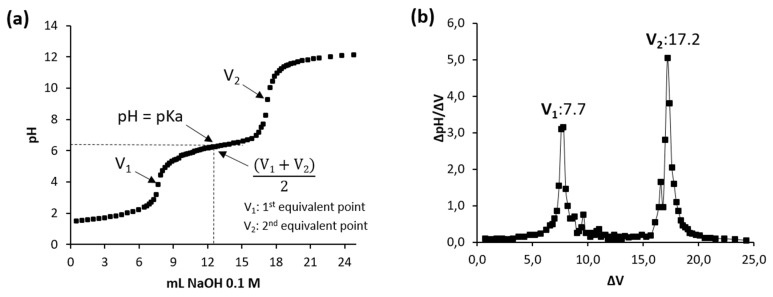
(**a**) Potentiometric titration of Chitosan-CGNA at pH 1.0–12.0; (**b**) first derivative curve of potentiometric tritation.

Potentiometric titration was used to determine the equivalent points and pK_a_ value of chitosan. In brief, 0.2 g of chitosan was dissolved under stirring in 20 mL of HCl (0.1 M) for 48 h to obtain a solution of 1% *w*/*v*. Then, aliquots of 0.5 mL of NaOH solution at 0.1 M (titrant) was added to the solution and the pH obtained was recorded using a pH meter (Hanna Instruments, Woonsocket, RI, USA). The titration was stopped when the pH value of the solution reached a value of 12. Then, the titration curve of pH *v*/*s* volume of NaOH consumed was plotted. At the graph of measured pH versus volume of NaOH consumed, each inflexion point was determined, where V_1_ corresponds to the first equivalent point, and V_2_ is the second equivalent point. Finally, pK_a_ value for Chitosan-CGNA was estimated as shown in Figure 2.

### 2.8. Calculation of the Protonation Degree of Chitosan

The protonation degree (DP) as a function of pH was calculated according to the Equation (5). The protonation degree (DP) can be defined as:

(4) $$\mathrm{DP}=C_{{\mathrm{NH}}_{3}^{+}–R}/C$$

where C_NH3_^+^_–R_ is the concentration of protonated amine groups and C is the total concentration of chitosan in solution. Based on the above equation, the relationship between the DP and pK_a_ is:

(5) $${\mathrm{pK}}_{a}=\mathrm{pH}-\log\left(\left(1-\mathrm{DP}\right)/\mathrm{DP}\right)$$

where pK_a_ corresponds to the value obtained from the potentiometric titration for chitosan and DP corresponds to the degree of protonation, leaving an equation with DP as a function of pH.

### 2.9. X-ray Diffraction (XRD)

X-ray diffraction spectra for commercial chitosan and Chitosan-CGNA were collected using an X-ray diffractometer (Bruker D2 PHASER, Berlin, Germany). The radiation was generated from a Cu Kα (λ = 1.789 Å) source. The diffraction data were collected from 10° to 100°, where 2θ is the angle of incidence of the X-ray beam on the sample.

### 2.10. Scanning Electron Microscopy (SEM)

Morphology of Chitosan-CGNA was determinated by SEM (SU3500, Hitachi, Tokyo, Japan), at acceleration voltages of 30 kV, 20 Pa vacuum and Backscattered Electron imaging (BSE).

### 2.11. Estimation of Water-Solubility

The pH dependence of water solubility of chitosan was evaluated from turbidity. The estimation of the solubility in water was carried out according to Qin et al. [15] with some modifications. Thus, the sample (100 mg) was dissolved in 100 mL of different solutions: 1% (*w*/*v*) acetic acid, 1% (*v*/*v*) lactic acid, 1% (*v*/*v*) HCl. Following stepwise addition of concentrated NaOH, the transmittance of the solution was recorded on a Multi-Detection Microplate Reader (Bio Tek Synergy HT, Winooski, VT, USA) at 600 nm.

### 2.12. DPPH Radical Scavenging Ability

The DPPH radical scavenging activity was measured according to Huang and Tsai [8] with minor modifications. An aliquot of 0.5 mL of each sample solution (2–10 mg/mL) was mixed with 0.5 mL of DPPH (0.1 mM) in 95% ethanol. The mixture was shaken thoroughly and kept in darkness at room temperature for 30 min. The absorbance was measured using a Multi-Detection Microplate Reader (Bio Tek Synergy HT, Winooski, VT, USA) at 517 nm. Ascorbic acid was used as a positive control. The DPPH radical scavenging activity was calculated as follows:

(6) $$\mathrm{DPPH}\;\mathrm{radical}\;\mathrm{scavenging}\;\mathrm{activity}\;\left(\%\right)=\left(1-A_{1}/A_{0}\right)\times 100$$

where A_0_ is the absorbance of the control (distilled water) and A_1_ is the absorbance of the sample.

### 2.13. Minimum Inhibitory Concentration (MIC) Determination

The bacterial strains were obtained from CECTA-USACH collection. To obtain the bacterial stock cultures, a single colony from each bacterial strain were isolated and growth at 35 °C ± 2 (18 h, 120 rpm) in an appropriated broth, Brain Heart Infusion broth (BHI, Merck, Germany) to *E. coli* (ATCC 25922), *S. aureus* (ATCC 25923) and *E. faecalis* (ATCC 29212); Buffered Peptone Water (Merck, Darmstadt, Germany) to *S. typhimurium* (ATCC 14028); Tryptone Soya Yeast Extract Broth (Merck, Darmstadt, Germany) to *L. monocytogenes* (ATCC 13932); CASO broth (Merck, Darmstadt, Germany) to *P. aeruginosa* (ATCC 15442). To the MIC determination a subculture of each bacterial strains was obtained by inoculum from stock culture in Muller Hinton broth (Merck, Darmstadt, Germany) pH 5.5 at 35 °C ± 2 (18 h, 120 rpm). To the MIC determination the Chitosan-CGNA and commercial chitosan were dissolved in 0.1 M acetic acid solution.

For MIC determination, the micro-dilution broth method was used [16,17]. Overnight subculture in MHB (pH 5.5) of each bacterium were adjusted to 10^7^ CFU/mL, and two-fold serial dilutions of Chitosan-CGNA and commercial chitosan were prepared in sterile MHB (pH 5.5). In a 96 well microplate, 20 µL of each bacteria subculture was aggregated to 120 µL of different concentration of Chitosan-CGNA or commercial chitosan and incubated for 20 h at 35 °C with 15 min of shaking at 45 min intervals. The MIC was determined following the Optic Density at 600 nm (OD_600_) to each bacterium in a Microplate Reader Synergy^TM^ HTX (Figures S1 and S2). In all the assays was included a culture in MHB (pH 5.5) of the respective bacteria without chitosan as negative control, and dilutions of chitosan in sterile MHB (pH 5.5) without bacteria as blank. All experiments were done in triplicate. The MIC endpoint was the lowest concentration of chitosan where no OD_600_ increase was detected after 20 h of incubation.

### 2.14. Minimum Bactericidal Concentration (MBC) Determination

To the MBC determination to Chitosan-CGNA and commercial chitosan, an aliquot of 50 μL from each well of the incubated microplate for the MIC determination to each bacterium was seeded on Nutrient Agar (Merck, Darmstadt, Germany) and incubated for 24 h at 35 °C.

The MBC was defined as the lowest concentration of chitosan that prevented any visible bacterial growth on the plate after 24 h incubation at 35 °C.

### 2.15. Fourier Transform Infrared Spectroscopy (FTIR)

FTIR spectra of chitosan samples were recorded in an IRPrestige-21 spectrometer (Shimadzu Corporation Pte. Ltd., Kyoto, Japan) according to Opazo-Navarrete et al. [18]. For each measurement, a total of 128 scans were collected at 4 cm^−1^ resolutions from 4000 to 400 cm^−1^, measurements were taken at room temperature using approximately 2 mg of each sample. The samples were placed on the surface of the ATR crystal and pressed with a flat-tip plunger until spectra with suitable peaks were obtained. The measurements were repeated three times and averaged to reduce baseline effects. Between measurements, the ATR was purged with water until no chitosan signal was detectable, and then the spectra were manipulated. The spectra were smoothed with a 9-point Savitsky–Golay function and normalized second-derivative spectra were obtained with the IR-solution software version 1.10 (Creon Lab Control AG, Shimadzu Corporation Pte. Ltd., Kyoto, Japan). Finally, spectra were cut and analyzed.

### 2.16. Statistical Analysis

Data were expressed as means ± standard deviations. All data were subjected to a t-student test to compare the means between the two independent groups. Differences were considered statistically significant at *p* < 0.05.

## 3. Results and Discussions

### 3.1. Physicochemical Characterization of Chitosan

#### 3.1.1. Degree Deacetylation (DD)

The DD of chitosan samples (hereafter referred as Chitosan-CGNA and commercial chitosan, respectively) are shown in Table 1. The chitosan obtained from *P. pugnax* crayfish exoskeleton reached a DD value of 91.5%, which was significantly (*p* < 0.05) higher than commercial chitosan (85.1%). The variations observed in the DD between Chitosan-CGNA and commercial chitosan (control) can be attributed to the purification methods and the reaction conditions used in this study. According to Rasweefali et al. [19], the factors that affect the DD of chitosan are the isolation method of chitin, source of extraction, species, alkaline concentration, reaction time, and temperature.

Yuan et al. [20] reported that the DD of chitosan is generally controlled by factors involved in the process of the native polymer with an alkali such as processing time, and temperature to obtain the highest DD (>90%) materials. Ali et al. [21], reported a DD of 92% for mud crab chitosan using 55% NaOH at 110 °C. Similar conditions (60% NaOH at 120 °C) were used in the present study for obtaining chitosan from *P. pugnax* crayfish exoskeleton. According to Rasweefali et al. [19], NaOH concentration up to 60% is suggested for obtaining chitosan with a significantly high DD. Hence, the high purity of this cationic biopolymer was influenced by the process conditions applied to obtain the chitin and, subsequently, chitosan.

The chitosan DD is one of the most important chemical parameters that determines many physiochemical and biological properties, and it influences the performance in many of its applications [22]. For instance, chitosan with a DD of 85–95% is defined as a high DD, which confers good solubility in water. While a DD of 55–70% is defined as a low DD of chitosan, being almost completely insoluble in water [23]. Most commercial chitosan samples show average DD values of 70–90%. For some specific biological applications, chitosan with higher DD values (>95%) can be produced by further deacetylation steps, which not only increases the cost of production, but often leads to partial depolymerization [24]. According to the abovementioned features, the Chitosan-CGNA could be considered as chitosan with a high purity and could be applied, for instance, in biomedicine applications.

#### 3.1.2. Molecular Weight of Chitosan

In this study, the molecular weight (Mw) of Chitosan-CGNA was 589.43 kDa, while M_W_ of commercial chitosan was lower (536.58 kDa) (Table 1). The slight differences in the M_W_ could be attributed to the conditions and reaction times adopted to obtain each chitosan sample. Younes et al. [25] reported that the factors affecting the average Mw of chitosan are the concentration of alkali, extraction process, raw material, DD, and solubility. Based on the range of its Mw, chitosan can be classified into three different types, namely, high-molecular-weight chitosan (>700 kDa), medium-molecular-weight chitosan (150–700 kDa), and low-molecular-weight chitosan (less than 150 kDa) [26]. Therefore, according to these classifications, the Chitosan-CGNA obtained in the present study would be classified as medium-molecular-weight chitosan. In general, the Mw of chitosan is one of the most significant parameters to determine its applications [19]. For instance, chitosan with medium Mw possesses anti-cancer activities, and chitosan with low Mw has antitumor, antioxidant, and antibacterial properties [19]. To date, high-molecular-weight chitosan and medium-molecular-weight have been widely used for bioplastics, flocculation, thin films and biofiber applications [26].

#### 3.1.3. Protein and Nitrogen Content

The protein and nitrogen content of chitosan samples are shown in Table 1. When the Chitosan-CGNA sample was compared with the commercial sample, similar nitrogen content was observed. The nitrogen content of Chitosan-CGNA (7.60 ± 0.1%) was slightly lower than commercial chitosan (7.74 ± 0.07%) (Table 1). The chitin and chitosan nitrogen generally ranged from 5 to 8%, making them more attractive to several industrial applications [19]. Regarding protein content, both Chitosan-CGNA and commercial chitosan presented a similar value (0.5%). These results indicate that the method used allows obtaining a product highly deacetylated and deproteinized.

#### 3.1.4. Solubility

The Chitosan-CGNA sample showed a solubility value of 98.50% in acetic acid (1%), and this result is similar to commercial chitosan under the study (98.95%). The solubility and DD are important properties determining the quality of the chitosan. The factors that can influence the chitosan solubility are reaction time, temperature, size of the product, alkaline concentration and a lower deacetylation rate. Moreover, the amino groups are protonated in acid media (pK_a_ lower than 6.2) leading to the solubility of chitosan [19].

#### 3.1.5. Intrinsic Viscosity

The intrinsic viscosity values are shown in Table 1. The viscosity of Chitosan-CGNA and commercial chitosan were 401.46 and 385.69 mL/g, respectively. Various parameters can affect the intrinsic viscosity of chitosan, such as concentration, molar mass, solvent, temperature, shear rate, chemical structure of the polymer and DD [27]. In the case of chitosan in acidic media, the degree of dissociation of the ionic groups also is an important factor that must be considered. The presence of ionic groups in their structures leads to expansion of the polymer chains due to the electrostatic repulsions, causing an increase in viscosity [27]. It should be noted that some biological properties of chitosan depend on its viscosity [28]. For instance, Cho et al. [29] reported that the antimicrobial activity of chitosan increased with decreasing viscosity.

#### 3.1.6. Ash Content

The determination of ash content is an important parameter to evaluate the effectiveness of the demineralization process of chitosan. Thus, ash content of Chitosan-CGNA and commercial chitosan were determined in this study. It is important to mention that the level of demineralization and deproteination determines the purity of chitosan, which affects its biological properties such as immunogenicity, biocompatibility and biodegradability [30]. According to Ssekatawa et al. [30], chitosan with an ash content lower than 1% possesses superior biological properties and is recommended for biomedical applications. The chitosan obtained in this study presented an ash content of 0.11% and this value is fit for medical applications. In addition, the variation observed in the ash values between Chitosan-CGNA and commercial chitosan could be attributed to the differences in the demineralization conditions used for each chitosan (e.g., HCl concentration and solid-to-solvent ratio).

### 3.2. Potentiometric Titration and Protonation Degree of Chitosan

Potentiometric titration is widely used to investigate polyelectrolyte behaviour in solution. For instance, previous studies have reported the titration and precipitation behaviour of chitosan during neutralization [31]. Figure 2a shows the variation of pH values as a function of NaOH added volume for chitosan solution. It can see that the potentiometric curve shows two inflexion points, which correspond to equivalent points (indicated in the Figure 2a). The first point (V_1_) corresponds to the neutralization of the excess of HCl (H^+^ + OH^−^ → H_2_O). While the second point (V_2_) is attributed to the protonation of amine groups of GlcN residues of chitosan. It should be noted that the determination of the first derivative helps in a precise reading of V_1_ and V_2_ (equivalent points) (Figure 2b). According to Pérez-Álvarez et al. [32], in acidic pH’s primary amino groups are protonated, and as NaOH is added during titration, they are neutralized (–NH_3_^+^ + OH^−^ → –NH_2_ + H_2_O) and their concentration can, thus, be quantified. In addition, the chitosan pK_a_ value (6.22) was obtained from the potentiometric titration curve. This pK_a_ value is similar to that registered by other chitosan [33].

**Figure 3:**
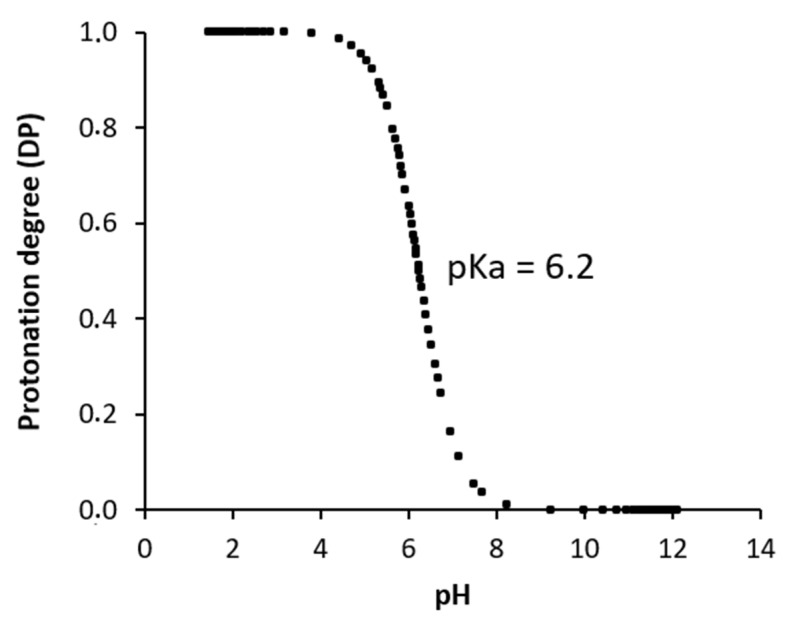
Protonation degree curve of chitosan at pH 2.0–12.0.

On the other hand, potentiometric titration was performed to evaluate the pH-dependent ionization degree of chitosan (Figure 3). The dissociation degree (DP) of chitosan depending on pH was calculated from Equation (5) and it was calculated using pK_a_ = 6.2. The degree of chitosan chain ionization progressively decreased with an increase in pH from 2 to 12, with a large drop observed between pH 6.0 and 7.5 (Figure 3). This decrease would, theoretically, result in a progressive reduction of the electrostatic repulsion between chitosan chains promoting their flexibility and agglomeration at a higher pH [34]. The pK_a_ is the pH of the inflexion point of the curve (point indicated in the Figure 3). The reason for the pH-dependency is the protonation/deprotonation of the –NH_3_^+^ group in chitosan.

### 3.3. Fourier Transform Infrared (FTIR) Spectroscopy

**Figure 4:**
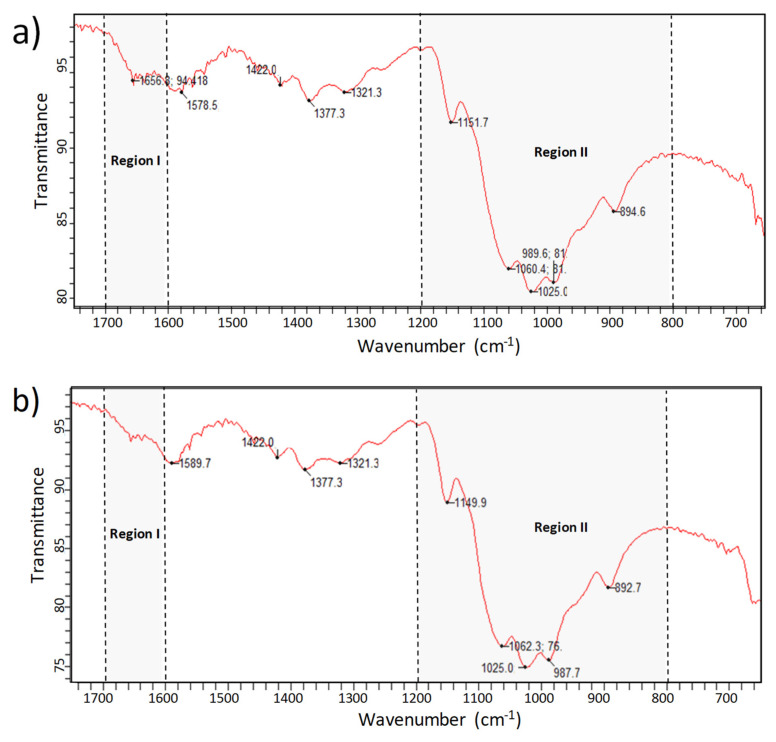
FTIR spectrum of (**a**) Chitosan-CGNA; and (**b**) commercial chitosan.

Figure 4 shows the FTIR absorption spectra of Chitosan-CGNA and commercial chitosan. The spectral range between 4000 and 1750 cm^−1^ has been omitted because chitosan does not present vibrational bands in that range. Both spectra of chitosan powders show the characteristic bands of chitosan previously reported by other authors [35]. For the analysis, the spectra have been separated in two characteristics regions; Region I (1600–1700 cm^−1^) which is traditionally assigned to proteins, and Region II (1200–800 cm^−1^) which is typically assigned to polysaccharides. Regarding the Region I, the spectra did not show differences between Chitosan-CGNA and commercial chitosan. Thus, both samples showed the characteristic amide I band at 1656 cm^−1^, which occurs at similar wavelengths in polyamides and proteins [36,37]. It is commonly assigned to stretching of the C=O group hydrogen bonded to N–H of the neighbouring intra-sheet chains [37].

Another characteristic band of –NH_2_ bending found in chitosan is at 1580 cm^−1^. In both samples this band shifted to 1578 cm^−1^ for Chitosan-CGNA and 1589 cm^−1^ for commercial chitosan. The other characteristic bands of CH_n_ groups at 1420 cm^−1^ (CH_2_ deformation), 1380 cm^−1^ (–CH_3_ symmetric deformation), and 1320 cm^−1^ (amide III and CH_2_ wagging) were shifted in both samples to 1422 cm^−1^, 1377 cm^−1^, and 1321 cm^−1^, respectively.

Meanwhile, the absorption bands in the Region II (1200–800 cm^−1^) show slight differences. This region belongs to the glycosidic ring, in particular, the band at 1156 cm^−1^ corresponds to the glycosidic linkage. This characteristic chitosan band was shifted to 1151 cm^−1^ for Chitosan-CGNA and 1149 cm^−1^ for commercial chitosan. Therefore, the FTIR spectra exhibited similar bands, suggesting that both chitosan samples share similar chemical compositions.

### 3.4. X-ray Diffraction (XRD)

**Figure 5:**
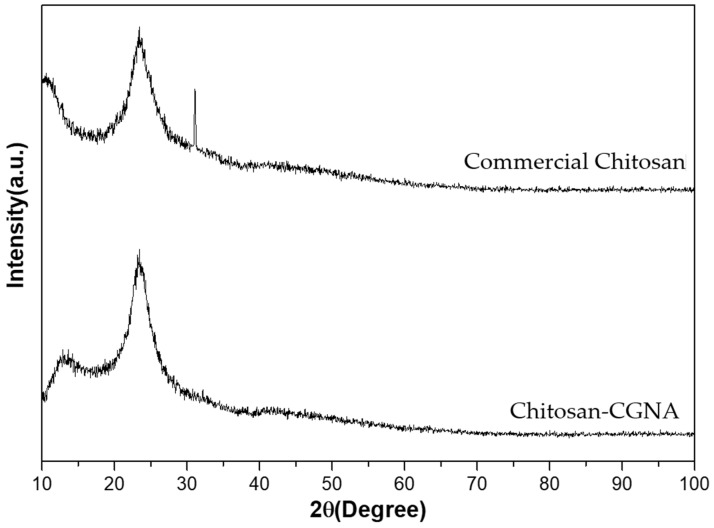
XRD pattern of Chitosan-CGNA and commercial chitosan.

X-ray diffraction spectra of Chitosan-CGNA and commercial chitosan are shown in Figure 5. The XRD pattern of Chitosan-CGNA exhibited its characteristic crystalline peaks at 2θ = 12.8° and 23.4°, whereas the commercial chitosan exhibited crystalline peaks at 2θ = 10.5°, 23.4°, and 31.1°. These results show that commercial chitosan has a higher crystalline degree. The reason for having different characteristic peaks might be caused by the source of chitin, as suggested by Kucukgulmez et al. [38] According to Leceta et al. [39], chitosan is a partially crystalline polysaccharide due to its regular chain. The reflection around 10° can be attributed to crystal forms I and the strongest reflection at 20° to crystal forms II. Pastor et al. [40] repoted that these two phases correspond to crystalline (less hydrated and harder) zones dispersed in an amorphous (more hydrated and softer) zone and describe the development of crystallinity in chitosan matrices due to the formation of hydrogen bonds between chains.

### 3.5. Scanning Electron Microscopy (SEM) Analysis

**Figure 6:**
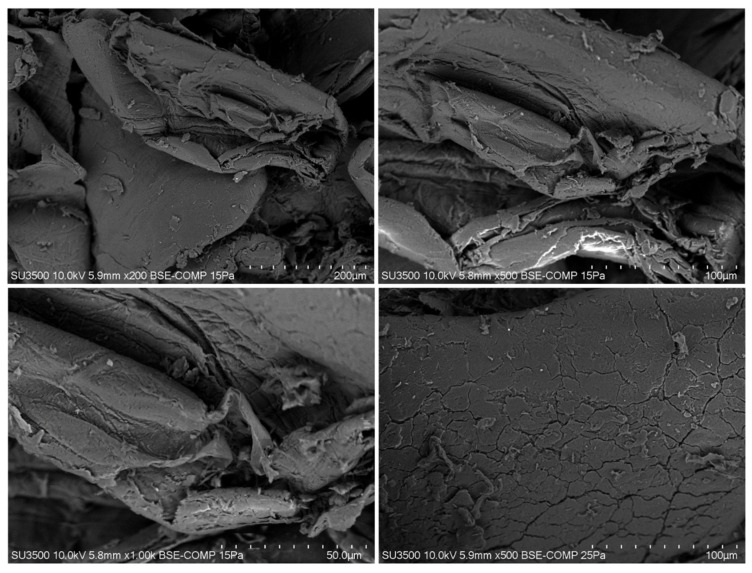
Scanning electron microscopy (SEM) of chitosan extracted from crayfish exoskeleton (*Parastacus Pugnax*).

The morphology of Chitosan-CGNA was studied by SEM at different magnifications and different areas of chitosan (Figure 6). Chitosan-CGNA showed aggregated flakes with a dense and firm structure and without porosity. When the magnification became higher in some parts of chitosan, crumbling flakes were observed with a fibrous structure. In general, chitin and chitosan may be classified into three surface morphologies: (1) with porosity and microfibrillar structure, (2) without porosity or microfibrillar structure, and (3) with only a microfibrillar structure [41]. In addition, the surface morphology chitosan can vary according to the source or organisms.

### 3.6. Effect of pH and Type of Acid on Solubility of Chitosan Samples

**Figure 7:**
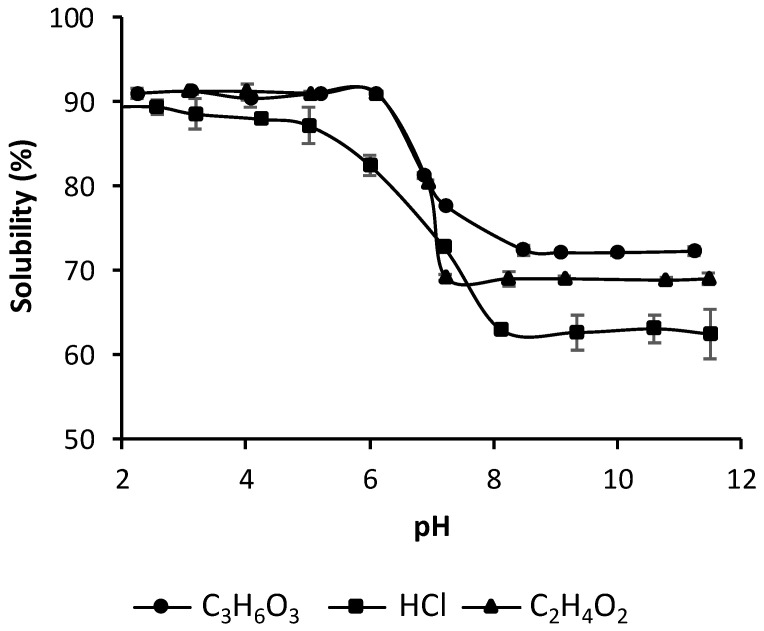
Solubility of Chitosan-CGNA with different acids at pH range from 2 to 11. Results are presented as means ± standard deviations (*n* = 3).

Chitosan is insoluble in water, aqueous bases, and organic solvents, but soluble in most organic acid solutions with pH < 6 [19]. Figure 7 shows the dependence of chitosan’s solubility on pH. The chitosan solubility obviously was dependent on pH variations and the type of acid used in this study (acetic acid, chlorhydric acid, and lactic acid). The results also showed that acetic and lactic acid solutions are a good solvent for chitosan dissolution, reaching the highest biopolymer solubility at pH ≤ 6, even though this was at a relatively higher pH value (pH > 7). The chitosan dissolution with chlorhydric acid was lower at the same conditions. Moreover, the difference of chitosan dissolution was even greater at pH values higher than 7, where the highest chitosan solubility was obtained with lactic acid followed by acetic acid and HCl. This indicates that the pH value is not the only dominant factor in the dissolution of chitosan. Consequently, it is concluded that a lower pH does not necessarily lead to the greater dissolution of chitosan, implying that the mechanism of chitosan dissolution is not the only protonation of the amino group, but may include interactions between chitosan and the acid as well. According to Chen et al. [42], the most used solvent for chitosan dissolution has been the acetic acid solution. It is a monoacid (with only one carboxylic group), and only serves as a proton donor in solution. In addition to acetic acid, there are many kinds of natural carboxylic acids, such as lactic acid, tartaric acid, and citric acid. Some binary and tertiary acids can react with two or more amino groups on separate chitosan chains, causing cross-linking of the chitosan into a large structure.

### 3.7. DPPH Radical Scavenging Ability

**Figure 8:**
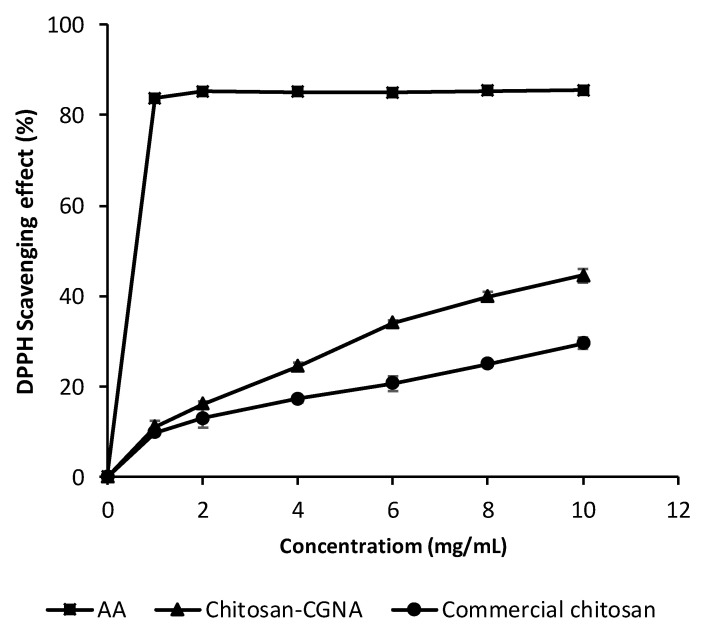
DPPH radical scavenging effect of chitosan samples. Results are presented as means ± standard deviations (*n* = 3). AA: ascorbic acid.

Figure 8 shows the DPPH radical scavenging activities of Chitosan-CGNA. Ascorbic acid was used as a control in this study. The chitosan sample shows different scavenging abilities in a dose-dependent manner. For Chitosan-CGNA, the DPPH scavenging abilities ranging from 0% to 44.57% at varying concentrations (0–10 mg/mL); this range was higher than the commercial chitosan sample (0–29.58%). According to Huang and Tsai [8], the DPPH radical scavenging effect of chitosan is related to the DD where a high value has a better antioxidant potential due to the number of free amino groups in its structure.

Therefore, the scavenging mechanism of chitosan could be explained by its active hydrogen atoms reacting with hydroxyl and superoxide anion radicals to form a stable macromolecule radical [8]. Kim and Thomas [43], reported that the scavenging mechanism of chitosan is related to the fact that the free radicals can react with the hydrogen ion from the ammonium ions (–NH_3_^+^) to form a stable molecule. The (–NH_3_^+^) has been formed by the amine group absorbing a hydrogen ion from the solution.

### 3.8. Antibacterial Activity

The chitosan antibacterial activity is influenced by several factors, such as temperature, pH, molecular weight (Mw), degrees of deacetylation (DD), chitosan concentration, type of microorganism, growth phase of the microorganism and culture medium composition [44,45,46].

Table 2 shows the Minimum inhibitory concentration (MIC) and the Minimum bactericidal concentration (MBC) of the Chitosan-CGNA and commercial chitosan against Gram-positive and Gram-negative bacteria. The results suggest that the antibacterial activity of Chitosan-CGNA and commercial chitosan have a slight trend toward Gram-positive than Gram-negative bacteria. This behavior agrees with No et al. [44] for chitosan samples between 746–470 kDa.

On the other hand, the results showed that the Chitosan-CGNA concentration to inhibit the *E. coli*, *S. typhimurium*, and *L. monocytogenes* growth was lower than the concentration of commercial chitosan. Whereas, the MIC and MBC of Chitosan-CGNA was higher than commercial chitosan with *S. aureus*, *E. faecalis* and *P. aeruginosa*. These results do not allow the suggestion that Chitosan-CGNA has a higher inhibitory or bactericidal activity than commercial chitosan but shed light on its potential use.

Previous studies performed with similar bacterial strains showed different MIC and MBC values [46,47,48]. According to Li and Zhuang [45], many factors can influence the antibacterial activity of chitosan. In this study, the Chitosan-CGNA showed a MIC value lower than other chitosan samples in *E. coli* [44,49], *S. aureus* [44,49], *P. aeruginosa* [49], and *L. monocytogenes* [44]. Whereas, with *E. coli*, *S. aureus*, and *S. typhimurium*, the MIC values of Chitosan-CGNA were higher than other reports [48]. The MBC values reported for Chitosan-CGNA were different to those previously reported; however, the MBC values of *S. typhimurim*, and *S. aureus* were equivalent to those reported by Kong et al. [48]

The differences in the MIC and MBC values between Chitosan-CGNA and others chitosan previously reported can be explained by different factors including pH, molecular weight, degrees of deacetylation, viscosity, all factors that influence the antibacterial activity of the chitosan [45,46,47,48].

The MIC and MBC determinations show that the Chitosan-CGNA has an antibacterial activity against all the bacteria used in this study. The inhibitory and bactericidal effect was evident at different concentrations of Chitosan-CGNA and depended on the bacterial species. These results allow the suggestion that Chitosan-CGNA could be used in applications against bacteria relevant to the food industry.

## 4. Conclusions

In the present study, chitosan from crayfish exoskeleton (*P. pugnax*) was produced and was physico-chemically characterized. The results revealed that chitosan extracted from the crayfish exoskeleton is a good quality biopolymer with similar properties to commercial chitosan. The chitosan had a medium molecular weight and a high DD. Moreover, the results suggest that DD could have largely influenced the physicochemical and biological properties of chitosan. Regarding the antioxidant activities, this study demonstrated the higher reducing ability and hydroxyl radical scavenging activity in Chitosan-CGNA than those in commercial chitosan.

In addition, Chitosan-CGNA showed antimicrobial activity against both Gram-positive and Gram-negative bacterium strains. Thus, this new ingredient could be used as a promising antimicrobial agent.

Hence, the present study suggests that the *P. pugnax* crayfish exoskeleton can be considered as a potential source to produce chitosan for different applications.

## Figures and Tables

**Table 1:** Physicochemical characterization of chitosan samples.

Physicochemical Properties	Chitosan-CGNA	Commercial Chitosan
Molecular weight (kDa)	589.43 ± 14.03 ^a^	536.58 ± 16.4 ^b^
Nitrogen content (%)	7.60 ± 0.10 ^a^	7.74 ± 0.07 ^a^
Solubility (%)	98.83 ± 0.22 ^a^	98.95 ± 0.35 ^a^
Intrinsic viscosity (mL/g)	401.46 ± 12.71 ^a^	385.69 ± 18.11 ^a^
Deacetylation degree (%)	91.55 ± 0.44 ^a^	85.11 ± 0.40 ^b^
Protein content (%)	0.5 ± 0.03 ^a^	0.5 ± 0.04 ^a^
pK_a_	6.22	6.50
Ash content (%)	0.11 ± 0.01 ^a^	0.14 ± 0.01 ^b^
Appearance	white flakes	white flakes

^a,b^ Different superscript letters in the same row indicate a significant difference between groups (*p* < 0.05).

**Table 2:** Minimum Inhibitory Concentration (MIC) and Minimum Bactericidal Concentration (MBC) of chitosan.

Bacteria	Chitosan-CGNA		Commercial Chitosan	
	MIC (mg/mL)	MBC (mg/mL)	MIC (mg/mL)	MBC (mg/mL)
*E. coli*	0.16	0.31	0.31	0.63
*S. typhimurium*	0.04	0.04	0.63	0.63
*P. aeruginosa*	0.04	0.04	0.02	0.02
*S. aureus*	0.04	0.04	0.005	0.02
*L. monocytogenes*	0.08	0.16	0.63	1.25
*E. faecalis*	0.04	0.04	0.02	0.15

## Data Availability

Not applicable.

## Supplementary Materials

The following are available online at https://www.mdpi.com/article/10.3390/polym13142304/s1, Figure S1: Minimum inhibitory concentration (MIC), Figure S2: Minimum Bactericidal Concentration (MBC).
