# Supplementary material for: Synthesis of New Chitosan from an Endemic Chilean Crayfish Exoskeleton (Parastacus Pugnax): Physicochemical and Biological Properties
Source: Polymers (Basel). 2021 Jul 14;13(14):2304. doi: 10.3390/polym13142304 (PMC8309378; doi:10.3390/polym13142304)
Supplement: Supplementary file 1 [file polymers-13-02304-s001.zip › polymers-1289844-supplementary.pdf]

Figure S1. MIC determination to Chitosan-CGNA and Commercial Chitosan

The graphics shows the absorbance (OD_{600}) for each bacteria incubated whit different concentrations of chitosan at 35°C during 20 h. The MIC is the concentration of chitosan where the OD_{600} go down near to zero.

P. aeruginosa

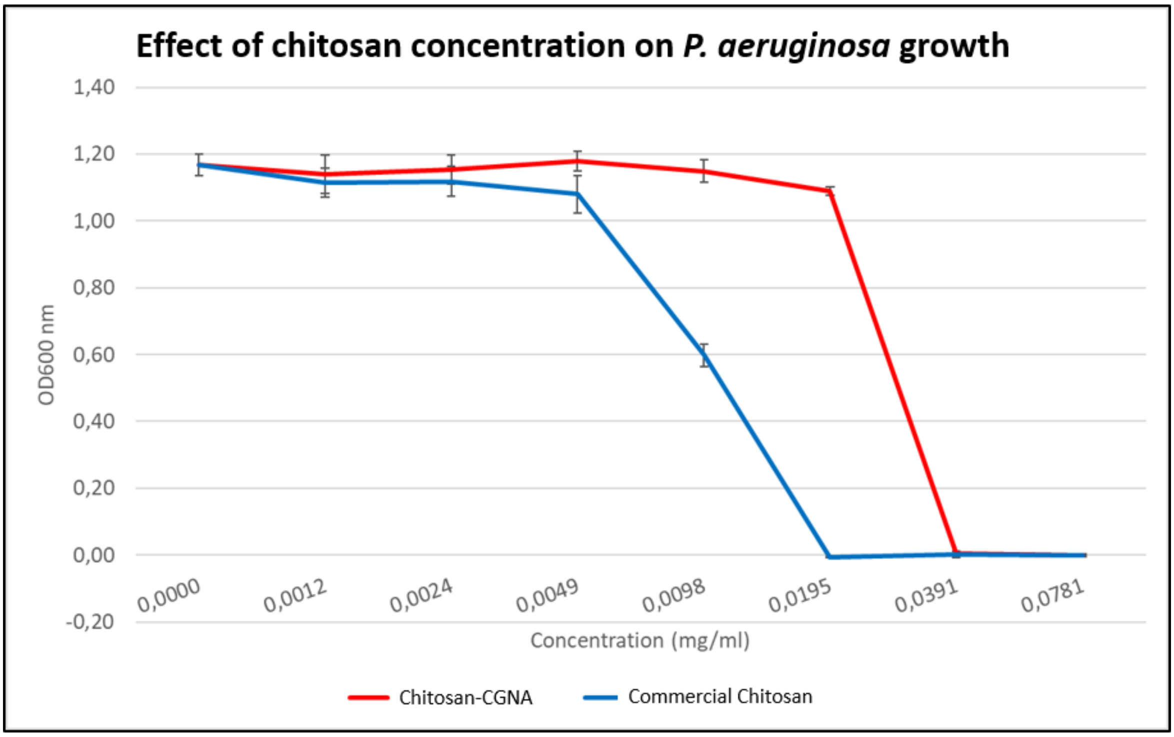

S. typhimurium

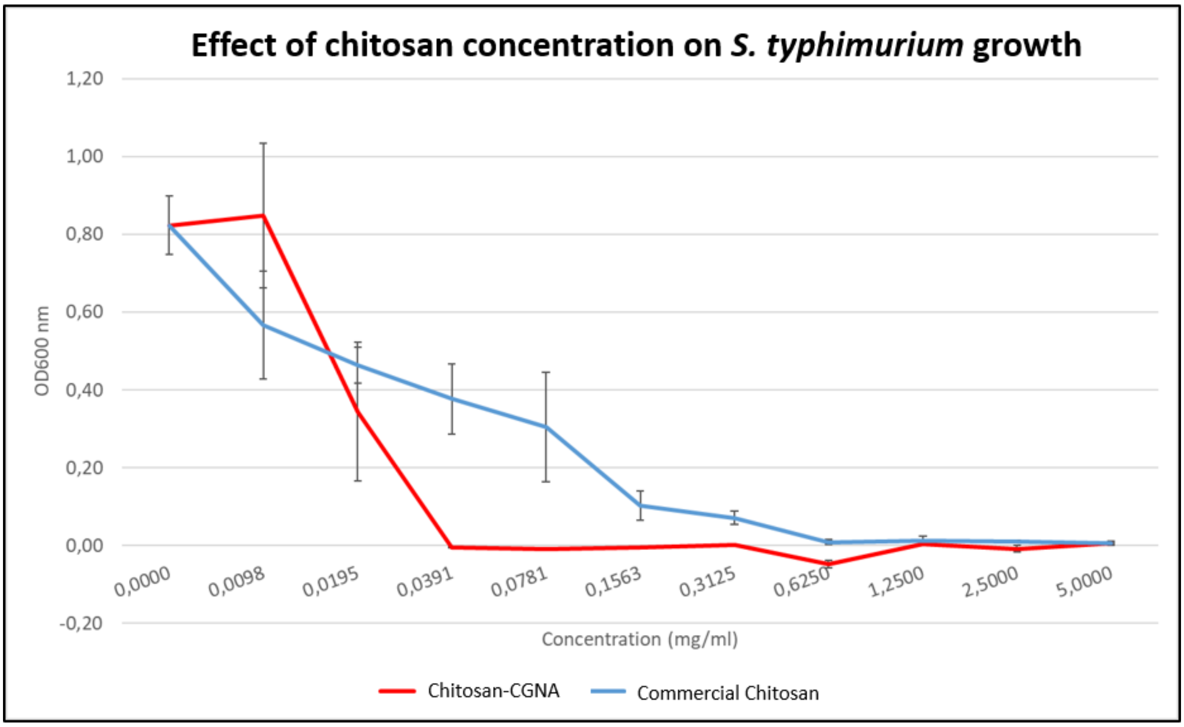

E. coli

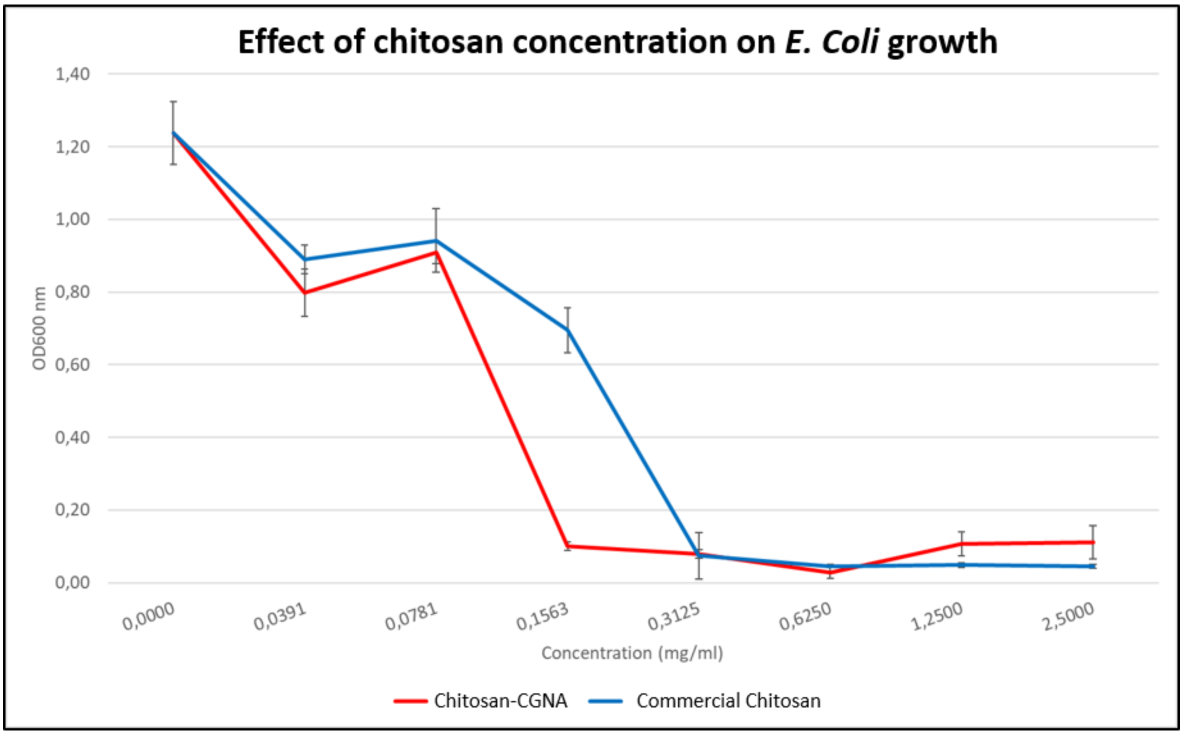

E. faecalis

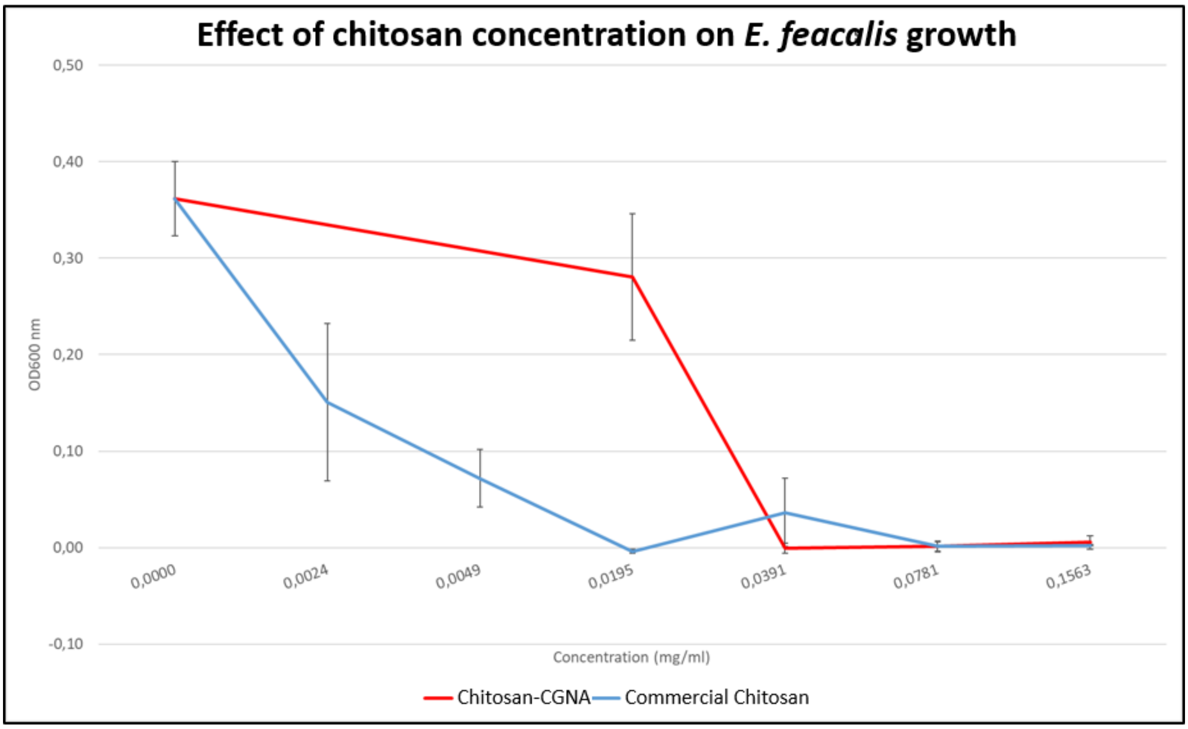

L. monocytogenes

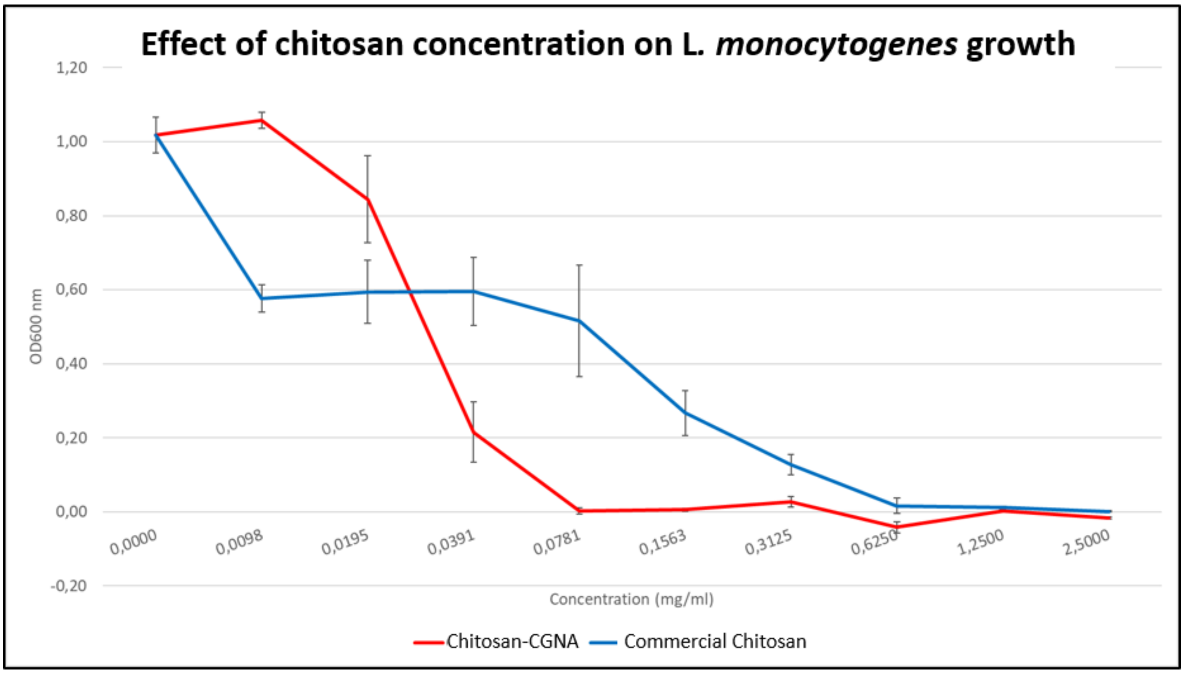

S. aureus

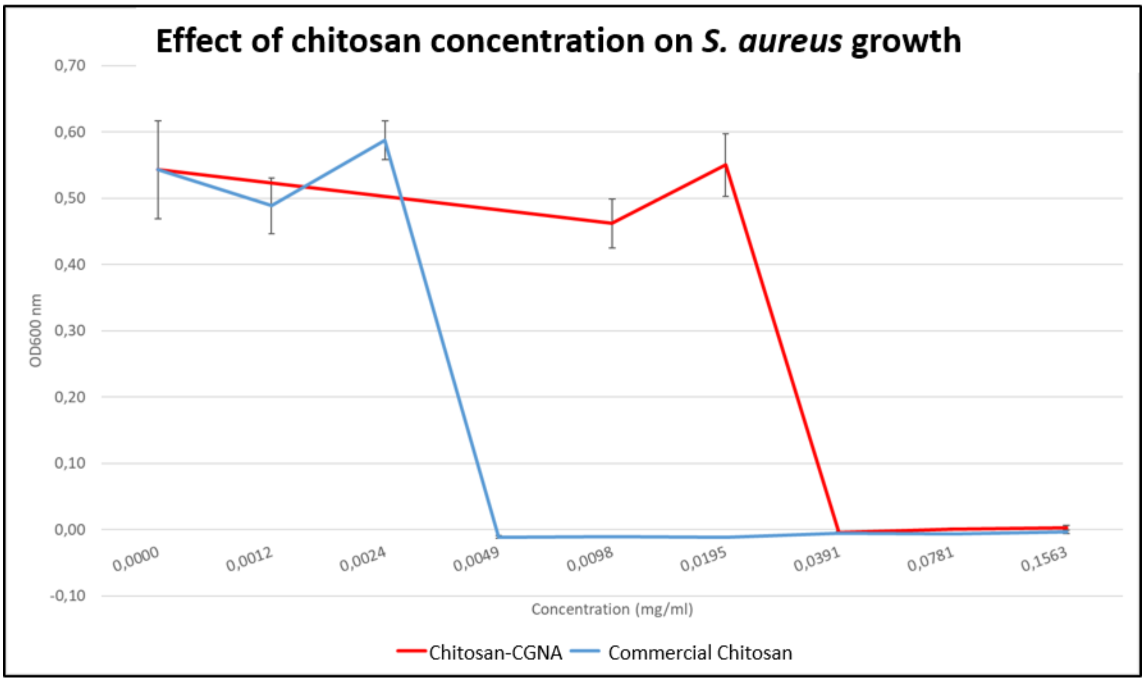

Figure S2. MBC Determination to Chitosan-CGNA and Chitosan-Commercial

Plates of bacteria previously incubated with different concentrations of chitosan at 35°C.

The MBC is the lower concentration of chitosan that did prevent any visible bacterial growth on plate after 24 h incubation at 35°C±2.

P. aeruginosa

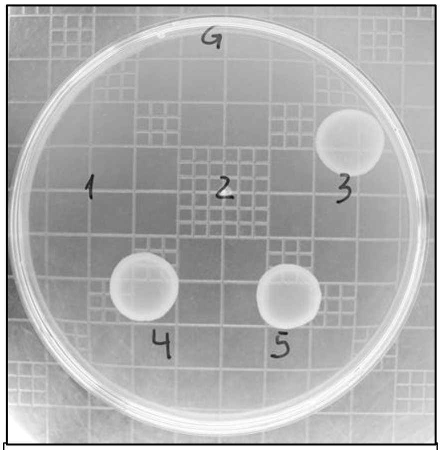

Chitosan-CGNA (mg/ml): 1) 0.08; 2) 0.04; 3) 0.02; 4) 0.01; 5) 0.005

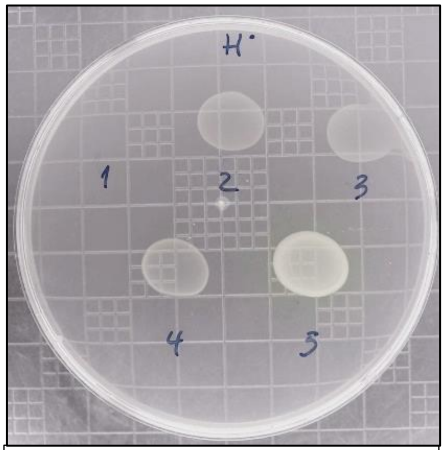

Chitosan-Commercial (mg/ml): 1) 0.02; 2) 0.01; 3) 0.005; 4) 0.002; 5) 0.001

S. typhimurium

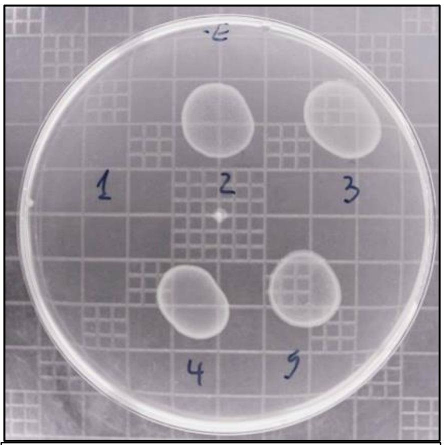

Chitosan-CGNA (mg/ml): 1) 0.04; 2) 0.02; 3) 0.01; 4) 0.005; 5) 0.002

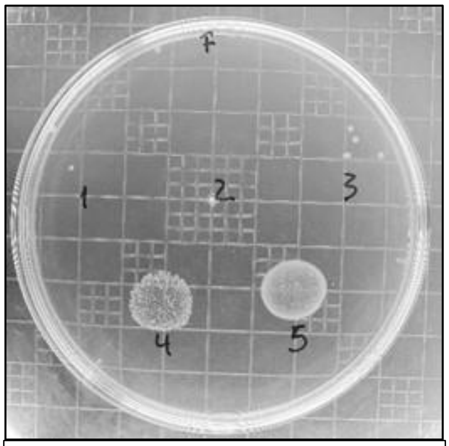

Chitosan-Commercial (mg/ml): 1) 1.25; 2) 0.63; 3) 0.31; 4) 0.16; 5) 0.08

E. coli

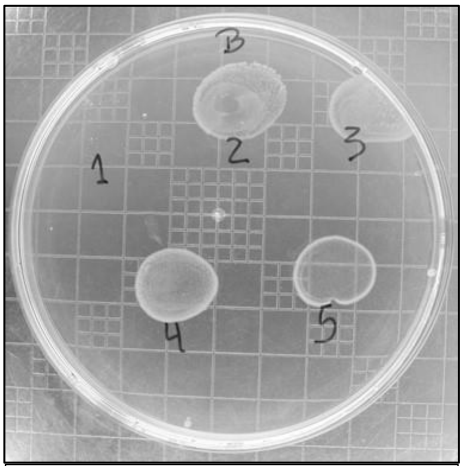

Chitosan-CGNA (mg/ml): 1) 0.31; 2) 0.16; 3) 0.08; 4) 0.04; 5) 0.02

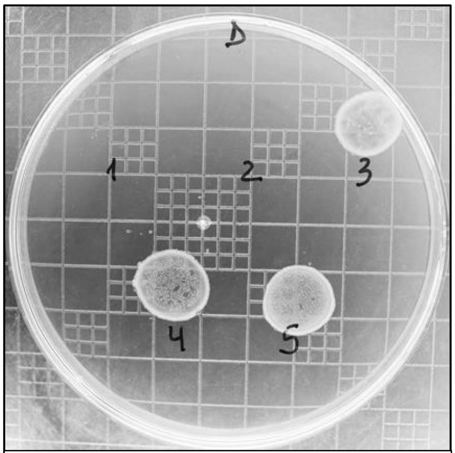

Chitosan-Commercial (mg/ml): 1) 1.25; 2) 0.63; 3) 0.31; 4) 0.16; 5) 0.08

E. faecalis

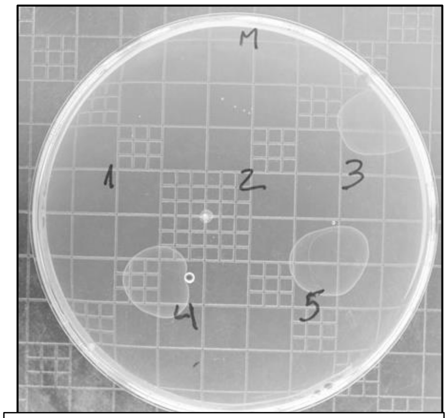

Chitosan-CGNA (mg/ml): 1) 0.04; 2) 0.02; 3) 0.01; 4) 0.005; 5) 0.002

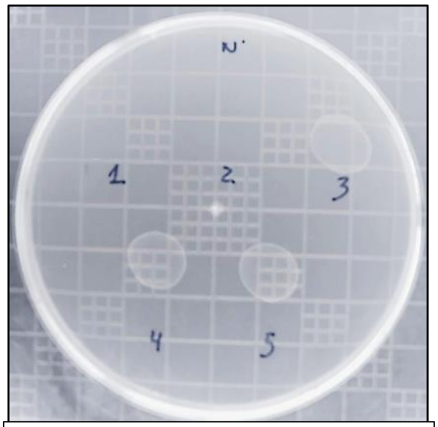

Chitosan-Commercial (mg/ml): 1) 0.31; 2) 0.15; 3) 0.08; 4) 0.04; 5) 0.02

L. monocytogenes

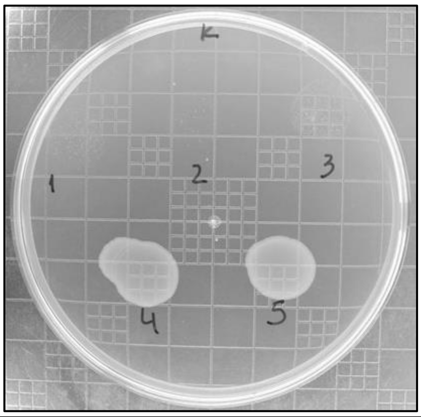

Chitosan-CGNA (mg/ml): 1) 0.31; 2) 0.16; 3) 0.08; 4) 0.04; 5) 0.02

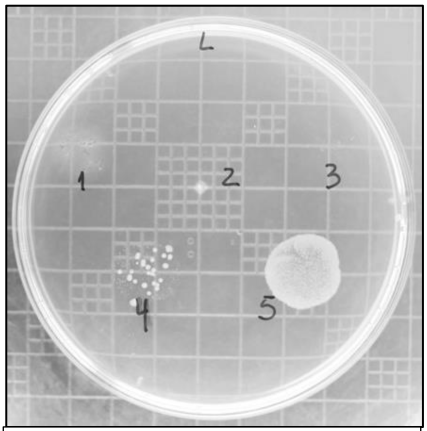

Chitosan-Commercial (mg/ml): 1) 5; 2) 2.5; 3) 1.25; 4) 0.63; 5) 0.31

S. aureus

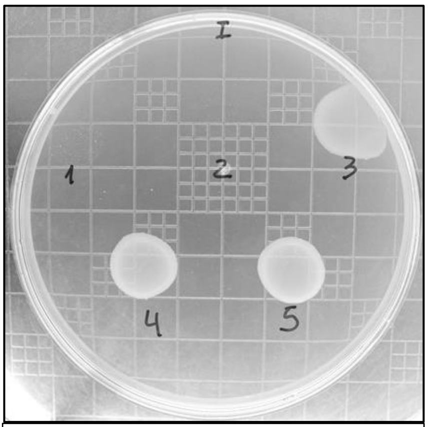

Chitosan-CGNA (mg/ml): 1) 0.08; 2) 0.04; 3) 0.02; 4) 0.01; 5) 0.005

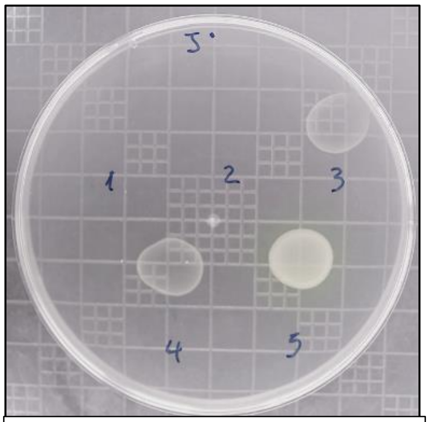

Chitosan-Commercial (mg/ml): 1) 0.04; 2) 0.02; 3) 0.01; 4) 0.005; 5) 0.002
